# The anticancer effects of curcumin and clinical research progress on its effects on esophageal cancer

**DOI:** 10.3389/fphar.2022.1058070

**Published:** 2022-10-28

**Authors:** Shimeng Wang, Xinliang Gao, Jialin Li, Shixiong Wei, Yifeng Shao, Yipeng Yin, Duo Zhang, Mingbo Tang

**Affiliations:** ^1^ Department of Plastic and Reconstructive Surgery, The First Hospital of Jilin University, Changchun, Jilin, China; ^2^ Department of Thoracic Surgery, The First Hospital of Jilin University, Changchun, Jilin, China

**Keywords:** curcumin, esophageal cancer, anticancer effect, bioavailability, gastrointenstinal

## Abstract

Esophageal cancer (EC) is a common tumor of the gastrointestinal system and a major threat to human health. The etiology and incidence of EC vary depending on the type of pathology. Owing to the unique physiological structure of the esophagus and the poor biological behavior of EC, the treatment modalities available are limited, and the prognosis of patients is relatively poor. Curcumin is a type of natural phytochemical belonging to the class of phenolic compounds. It exerts favorable anticancer effects on various cancers. A growing body of evidence indicates that curcumin suppresses tumor development and progression by inhibiting tumor cell proliferation, invasion, and migration, thus inducing apoptosis, regulating microRNA expression, reversing multidrug resistance, and inducing sensitivity to the therapeutic effect of chemoradiotherapy. Multiple cellular molecules, growth factors, and genes encoding proteins participating in different signaling pathways interact with each other to contribute to the complex and orderly anticancer effect. The efficacy and safety of curcumin have been established in preclinical studies for EC and clinical trials for other cancers. However, the low bioavailability of curcumin limits its clinical application. Therefore, the modification of curcumin analogs, the combination of curcumin with other drugs or therapies, and the use of novel nanocarriers have been widely investigated to improve the clinical effects of curcumin in EC.

## 1 Introduction

The esophagus forms the upper segment of the gastrointestinal tract and is an important part of the gastrointestinal system. Esophageal cancer (EC) is one of the most common gastrointestinal system malignancies worldwide, with approximately 604,000 new cases and 544,000 deaths reported in 2020 according to global cancer statistics (Sung et al., 2021). EC accounts for the seventh-highest incidence of cancer and ranks as the sixth-leading cause of cancer death globally (Sung et al., 2021). EC comprises two primary histological types, esophageal adenocarcinoma (EAC) and esophageal squamous cell carcinoma (ESCC), which differ in etiology and geography. The risk factors of EAC primarily include obesity, gastroesophageal reflux disease (GERD), and Barrett’s esophagus, and the increase in these risk factors in populations of high-income countries contributes to the high prevalence of EAC, accounting for approximately two-thirds of EC cases (Blot et al., 2018; Uhlenhopp et al., 2020). Barrett’s esophagus is characterized by the presence of a precancerous lesion and the induction of columnar metaplasia in the normal squamous epithelium owing to injury resulting from GERD in the distal esophagus (Clermont and Falk, 2018). However, in countries with a lower income, ESCC accounts for approximately 90% of patients with EC. Smoking, nitrosamine intake, dietary habits such as preference for hot food, pickled vegetables, and high alcohol consumption are important factors leading to ESCC (Uhlenhopp et al., 2020). The pathogenesis of EC has not been completely elucidated, and the occurrence of EC may result from the long-term interaction between genetic factors and environmental factors. However, despite extensive progress in research on the diagnosis and treatment of EC, its morbidity and mortality rates are still on the rise, and the 5-year survival rate remains low (Uhlenhopp et al., 2020).

The symptoms of EC are insidious in the early stage of the disease. When the symptoms of dysphagia and other discomforts become apparent, often, the disease has already been locally advanced. Very limited treatment options are available owing to the late diagnosis and poor biological behavior of EC. Currently, the treatment of EC is based on different stages and primarily includes endoscopic therapy, surgery, chemotherapy, and radiotherapy, among which endoscopic therapy is applied more commonly for early mucosal disease. For locally advanced EC, the significance of combining chemotherapy and radiotherapy with surgery has been established. For example, a meta-analysis including 17 trials and seven studies reported that neoadjuvant chemoradiotherapy or chemotherapy had a significant survival benefit over surgery alone for patients with EC (Sjoquist et al., 2011). A more recent clinical trial also had shown that patients with EC who received neoadjuvant chemoradiotherapy had higher overall survival rates than those who received only surgery (Shapiro et al., 2015). The technology used for EC treatment has made great advances, but the survival rate has not improved significantly, and the overall efficacy remains unsatisfactory. In addition, these treatment modalities also have certain negative effects, such as the various complications arising from surgery, drug toxicity from chemotherapy, and tissue damage from radiation. New therapeutic strategies need to be complemented by suppressing side effects and enhancing sensitivity to chemoradiotherapy to improve the outcome.

In recent times, dietary phytochemicals have received extensive attention in anticancer research. Some safe and nontoxic molecules isolated from plant products have been identified as potential anticancer agents. Curcumin (diferuloylmethane), a natural phenolic compound, is derived from the plant *Curcuma longa*. The rhizomes of this plant have been used extensively as a flavor supplement in Asian cuisine for centuries. Curcumin or 1,7-bis-(4-hydroxy-3-methoxyphenyl)-1,6-heptadieno-3,5-dione is composed of two ferulic acid residues linked by a methylene bridge. It is insoluble in water but soluble in several organic solvents (Gupta et al., 2013). The safety of curcumin is confirmed by its dietary consumption for centuries and evidence reported in clinical trials. Curcumin was also shown to be well tolerated and efficiently eliminated in humans (Cheng et al., 2001). A growing body of evidence indicates that curcumin possesses multiple pharmacological properties such as anticancer, antioxidant, and anti-inflammatory properties (Menon and Sudheer, 2007; Tomeh et al., 2019). In addition, the cancer-preventive potential and anticancer effect of curcumin remain important topics in cancer research. To this end, the effects of curcumin have been investigated in various cancers, such as prostate, colon, lung, esophagus, and breast cancers, among others (Allegra et al., 2017).

**FIGURE 1:**
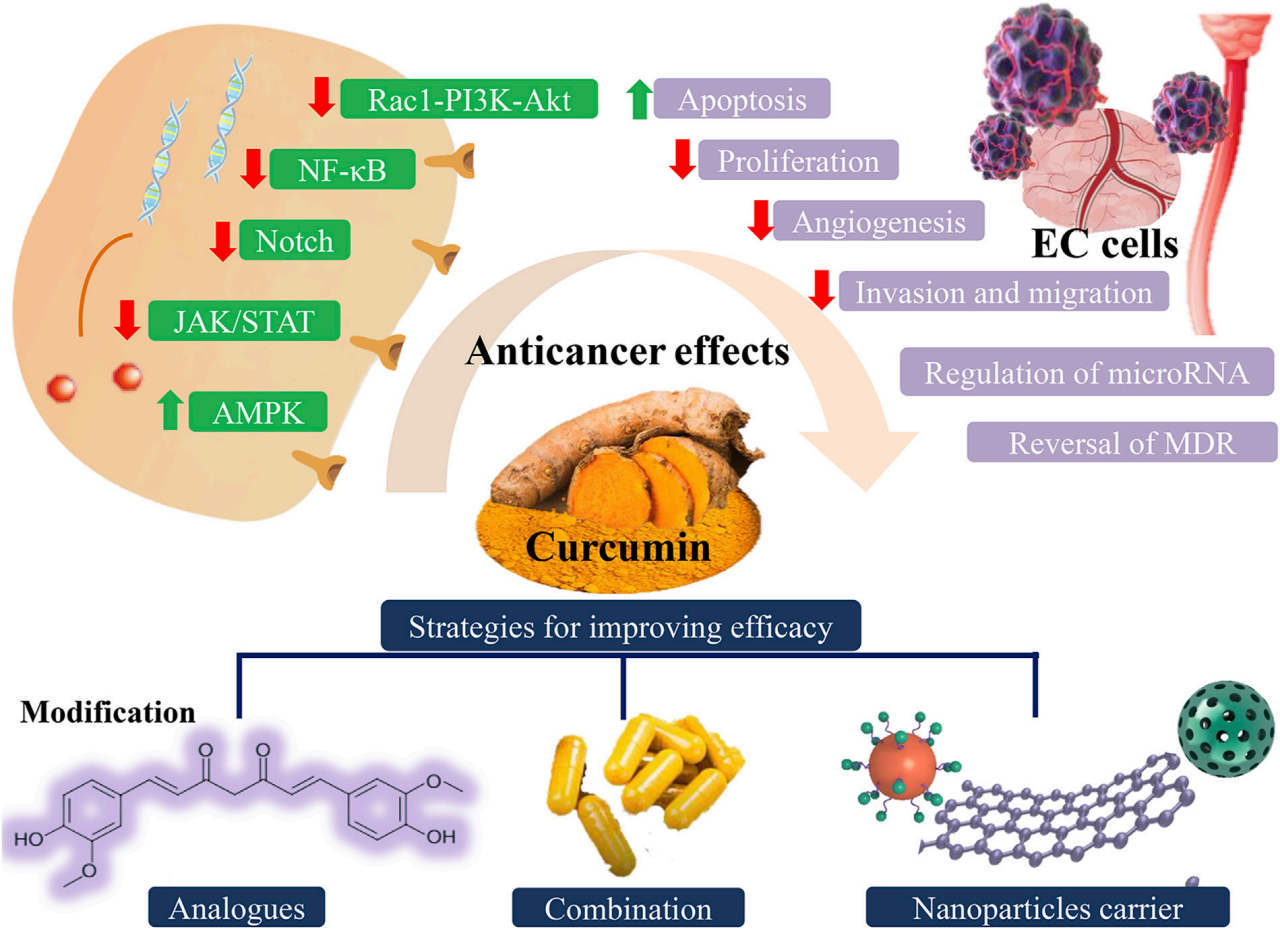
Different anticancer effects of curcumin involved in multiple signaling pathways and possible strategies for improving efficacy of curcumin. EC: esophageal cancer.

In this review, we summarize the anticancer mechanism of action of curcumin with respect to the effects reported *in vitro* in cancer cells, animal models, and clinical trials in the treatment of EC. Besides, we also highlight the importance of new analogs of curcumin, combination therapies, and novel delivery systems in EC, to enhance the clinical efficacy of curcumin. The mechanism of action of curcumin and strategies for improvement of efficacy are shown in Figure 1.

## 2 The anticancer mechanism of action of curcumin

The anti-tumor activity of curcumin can affect different types of cancer cells at multiple stages of tumorigenesis and cancer development. The anticancer mechanism of action of curcumin is complex and diverse and can be summarized from various aspects.

### 2.1 Growth inhibition and apoptosis induction

Cancer cells have the property of unlimited proliferation and induction of cell cycle arrest may be an effective way to suppress cell growth. A study reported that curcumin could trigger G2/M cell cycle arrest and cellular senescence in cervical cancer cell lines in a dose-dependent manner, leading to decreased cell survival (Wang et al., 2020). In addition to inhibiting cell growth, curcumin was also involved in the induction of cellular autophagy by promoting the accumulation of reactive oxygen species (ROS) (Wang et al., 2020). Excessive ROS levels can affect cell function and even cause cell death. Under the oxidative stress, autophagy is activated and cellular components are degraded.

Apoptosis, also known as programmed cell death, is often regulated by a highly ordered multigene process of active cell death. Abnormalities in apoptosis lead to uncontrolled cell proliferation and eventually to cancer development (Pistritto et al., 2016). Curcumin can induce the apoptosis of cancer cells and inhibit tumor growth through various mechanisms, thus achieving anti-tumor effects. Caspases play a critical role in apoptosis activation and execution. Apoptosis is a cascade reaction in which caspases are activated and perform their corresponding functions (Boice and Bouchier-Hayes, 2020). Curcumin was shown to induce apoptosis in EC cells by activating caspase 3 and continuously amplifying death signaling (Subramaniam et al., 2012). The promotion of apoptosis was characterized by the increased ratio of Bcl-2/Bax proteins. Reportedly, the mitochondria could be targeted by curcumin to induce apoptosis in cancer cells (Morin et al., 2001). Curcumin contributed to the management of the opening and closing of the permeability transition pore of the mitochondria. When curcumin induces cell apoptosis, it can increase the membrane permeability, leading to swelling of mitochondria. This causes the loss of the mitochondrial membrane potential and the blockade of ATP synthesis, thus triggering the death of cancer cells. Moreover, curcumin can activate apoptotic signaling by perturbing intracellular calcium homeostasis through the endoplasmic reticulum pathway in thyroid carcinoma cells (Zhang et al., 2018). The endoplasmic reticulum plays a key role in maintaining the stability of the cellular calcium content and the synthesis and processing of proteins. The increase in the cytosolic calcium content induced by curcumin can cause excessive endoplasmic reticulum stress, leading to the activation of the mitochondrial apoptotic pathway. Furthermore, the induction of a mitotic catastrophe may act as a distinct death mechanism, a type of non-apoptotic cell death (O'Sullivan-Coyne et al., 2009). These different targets of apoptosis induction may be promising therapeutic targets in the inhibition of cancer cells.

### 2.2 Inhibition of tumor cell invasion and migration

More patients with tumors are estimated to eventually die from the growth of metastatic tumors than from the primary tumor (Bergers and Fendt, 2021). The dispersal of tumor cells is the most dangerous process in tumor development. Epithelial-mesenchymal transition is characterized by an altered cell phenotype and enhanced cell migration and motility and is closely related to tumor metastasis (Yang and Weinberg, 2008). Tumor cells are dislodged from the primary site and bind to the extracellular matrix (ECM) *via* cell surface receptors and secrete proteases to promote cellular ECM breakdown to reach their metastatic sites (Seyfried and Huysentruyt, 2013). When tumor cells form clones in distant organs, they often cause serious damage to the organism.

Reduced intercellular adhesion is a morphological characteristic of malignancy and tumor metastasis and leads to cell detachment and destruction (Hirohashi and Kanai, 2003). Curcumin-treated esophageal cancer cells showed the promotion of cell-cell adhesion and cell-matrix adhesion, which indicated the antimetastatic effect of curcumin (Zheng et al., 2018). Ray et al. (2003) showed that the binding to ECM, the expression of cell receptors, and the activity of collagenase in melanoma cells can be reduced by treatment with curcumin. In addition, curcumin enhanced the expression of nonmetastatic gene 23 (Nm23), antimetastatic protein tissue inhibitor metalloproteinase (TIMP)-2, and E-cadherin, which inhibited the migration of tumor cells. In addition, Wroński et al. (2021) revealed a mechanism of bladder cancer progression, in which cancer cells activated matrix metalloproteinases (MMPs) and invaded the urothelial basement membrane. Curcumin can inhibit the aberrant activation of MMPs and maintain the integrity of the basement membrane, thus suppressing eventual cancer invasion into the bladder. The anti-metastatic effect of curcumin in the different phases of tumor invasion provides a therapeutic opportunity to prevent or reduce tumor metastasis.

### 2.3 Anti-angiogenesis

Angiogenesis is an important condition for tumor development and facilitates the entry of tumor cells into the circulation for metastasis and dissemination (Folkman, 2002). Multiple growth factors, such as tumor growth factor β (TGF-β), vascular endothelial growth factor (VEGF), and angiopoietin, among others, are involved in angiogenesis and exhibit mutual interaction.

Curcumin and its analogs could potentiate the anti-angiogenic effects in pancreatic cancer (PC) cell lines by inhibiting the secretion of VEGF, angiopoietin, and TGF-β *via* the suppression of nuclear factor kappa B (NF-κB) and hypoxia-induced factor-1α (HIF-1α) (Nagaraju et al., 2015). Similarly, a reduction in angiogenesis was also observed in colorectal cancer (CRC) cells treated with curcumin and its analogs (Rajitha et al., 2017). Cancer cells elicited the decreased secretion of HIF-1α and VEGF owing to the inhibition of NF-κB expression by curcumin. Hepatic stellate cells (HSCs) were capable of inducing angiogenesis and cell invasion in the progression of hepatocarcinoma (HCC) (Li et al., 2016). The downregulation of HIF-1α induced by curcumin could decrease tube formation and VEGF expression in HSC-induced HCC, thus reducing tumor angiogenesis and invasion (Shao et al., 2019). The authors further suggested that the anti-angiogenesis effect of curcumin was attributed to the expression of a downstream gene of HIF-1α, namely connective tissue growth factor (CTGF). These growth factors may be potential targets for the prevention of tumor angiogenesis and progression.

### 2.4 Regulation of microRNA (miRNAs)

MiRNAs are a class of evolutionarily conserved non-coding small molecule RNAs that range from 20 to 24 nucleotides in length and function as regulators in gene expression at the translational level (Bartel, 2004). MiRNAs play a role in cell proliferation, apoptosis, differentiation, cell cycle, and metabolism. Findings from numerous studies have shown that curcumin affects tumor development through the regulation of miRNAs.

MiRNA-21 (miR-21), known as a type of onco-miRNA, contributes to tumor growth and proliferation by targeting the PTEN/PI3K/Akt pathway (Zhang et al., 2012). Curcumin could induce apoptosis and suppress proliferation in gastric cancer (GC) cell lines by downregulating miR-21 (Qiang et al., 2019). Besides, the decreased expression of Akt and increased expression of PTEN indicated that the PTEN/PI3K/Akt pathway was negatively modulated by curcumin to inhibit tumor progression. In another study, curcumin and its analogs were shown to decrease cell viability and induce apoptosis in CRC cell lines by downregulating specificity protein family, which is dependent on activation by ROS (Gandhy et al., 2012). ZBTB10 and ZBTB4 acted as repressors of specificity protein family and were regulated by miR-27a, miR-20a, and miR-17-5p. Therefore, curcumin exert its anticancer effect by the regulation of these miRNAs.

Moreover, miRNAs play a role in cell migration and drug resistance. The loss of expression of the epithelial marker E-cadherin has been recognized as one of the most distinctive features of EMT (Perl et al., 1998). The overexpression of miR-200 resulted in the reduction of cell migration in HCC cell lines *via* the upregulation of E-cadherin and inhibition of EMT, with no effect on cell growth (Hung et al., 2013). Low levels of miR-200 expression were associated with a poor differentiation status of HCC cells, as determined based on the cell morphology and migratory capacity. Besides, an association exists between miRNA and drug resistance. Liang et al. (2013) compared the effect of curcumin on different HCC cell lines (HepJ5 and HepG2) and found that HepJ5 cells expressed less miR-200a/b and were more responsive to curcumin than HepG2 cells. The overexpression of miR-200a/b may make HCC cells more resistant to curcumin. In addition, an increase in apoptosis was observed in control HepJ5 cells compared with that in HepJ5 cells overexpressing miR200 a/b after curcumin treatment. MiR200 may be a helpful biomarker to assess the anti-apoptotic effect of curcumin. Furthermore, EMT-suppressive miRNAs (miR200b/c, miR-429, miR-141, and others) were activated by curcumin to induce EMT inhibition, which suppressed proliferation and triggered apoptosis in 5-fluorouracil (5-FU)-resistant CRC cells (Toden et al., 2015). The overexpression of miR-200c enhanced the sensitivity of curcumin and 5-FU through the suppression of EMT, thus promoting drug efficacy. The role of the miR200 family requires to be elucidated further, as it exhibits varied effects in different cell lines.

### 2.5 Reversal of multidrug resistance

Chemotherapy is an important modality in the clinical treatment of oncological diseases. The development of multidrug resistance (MDR) in tumor cells is one of the major causes of poor prognosis in patients owing to chemotherapy failure and an important challenge in clinical antitumor therapy. Therefore, the inhibition of MDR in cancer cells and improvement of the efficacy of chemotherapeutic drugs are urgent needs in the field. Besides exerting anticancer effects, curcumin also exhibits MDR reversal properties.

Tang et al. (2005) assessed the effect of curcumin on the MDR of vincristine (VCR)-resistant GC cell lines (Tang et al., 2005). Curcumin could enhance apoptosis induced by VCR in VCR-resistant cells, which indicates that it countered apoptotic resistance in these MDR cells. The MDR-reversing potential of curcumin was achieved *via* the downregulation of P-glycoprotein (P-gp) expression and activation of caspase-3. P-gp could transport unrelated substances, such as drugs, through plasma membranes to reduce the level of cytotoxicity. Curcumin was also shown to reverse MDR in PC cell lines by suppressing multi-drug resistance-associated protein 5 (MRP5) and thus reducing drug efflux (Li et al., 2011). Moreover, Shakibaei et al. (2014) revealed a novel mechanism of action of curcumin in reversing resistance to 5-FU in 5-FU-resistant CRC cell lines by inhibiting cancer stem cells (CSCs). Curcumin could sensitize cells to 5-FU and potentiate the anticancer effects of 5-FU in chemo-resistant cells. CRCs treated with both curcumin and 5-FU showed a significant reduction in the expression of CSC markers (CD133, CD44, and ALDH1) compared to CRCs treated with either, which suggests that curcumin exerts a strong chemosensitizing effect on CSCs. CSCs are subpopulations of the tumor cell population with the potential for renewal and contribute to tumor invasion (Huang et al., 2020). Conventional chemotherapeutic agents cannot destroy cancer cells owing to the drug resistance of CSCs. The functions of curcumin in drug resistance promote the clinical application of conventional chemotherapeutic drugs.

### 2.6 Sensitization of radiation therapy

Radiotherapy may be one of the preferred methods for patients with cancer who are not sensitive to chemotherapy. However, some patients are also resistant to radiotherapy. Novel radiosensitization approaches are needed to increase the responsiveness of patients to radiation. The application of curcumin as an adjuvant in radiotherapy has been shown to be effective.

Curcumin combined with gamma-radiation inhibited tumor growth in mice with a colon cancer xenograft through the downregulation of the NF-κB pathway (Kunnumakkara et al., 2008). The expression of the proliferation marker Ki-67, angiogenesis markers (CD31 and VEGF), and NF-κB-regulated genes decreased in tumor tissues, leading to the enhanced susceptibility of cancer cells to radiation and suppression of tumor growth. Liu et al. (2018a) also demonstrated that curcumin potentiated the anticancer effect of radiation in ESCC cell lines by inhibiting the NF-κB pathway. Treatment with curcumin in an irradiated environment promoted apoptosis induction in cancer cells and prolonged survival in xenograft mice. Moreover, Shehzad et al. (2013) revealed another mechanism by which curcumin improved the sensitivity of cancer cells to radiation therapy. Curcumin enhanced ionizing radiation-induced death in colon cancer cells by downregulating the pre-mRNA processing factor 4 kinase (Prp4K) and upregulating ROS. Prp4K is an antioxidant enzyme that protects cells against oxidative damage, and the overexpression of Prp4K conferred resistance to radiation in cancer cells.

## 3 Anticancer effect of curcumin in preclinical studies of EC and clinical trials of other cancers

### 3.1 *In vitro* studies

Preclinical *in vitro* studies have shown the efficacy of curcumin in the treatment of EC (Table 1). Accumulating evidence indicates that curcumin can affect different cellular and molecular signaling pathways involved in tumor development. Zhang et al. (2015) reported that curcumin could suppress cell growth and cause cell cycle arrest in the G2/M phase in esophageal Ec109 cells by activating the AMPK signaling pathway. Cancer cells can increase energy metabolism through glycolysis to promote their proliferation. AMPK plays an important role in cellular metabolic activities. The curcumin-mediated activation of AMPK was associated with a decrease in the expression of glycolytic enzymes, which reduced the energy demand of cancer cells and suppressed their proliferation. Another signaling pathway, the NF-κB signaling pathway, is involved in cancer cell proliferation, chemoresistance, and metastasis (Dolcet et al., 2005). NF-κB was shown to be a contributing factor in the progression of Barrett’s metaplasia to EAC (Hartojo et al., 2010). Barrett’s metaplasia samples showed lower expression of NF-κB and greater expression of the effector caspase compared to that in EAC. Besides, in esophageal Flo-1 and OE33 cell lines treated with curcumin, NF-κB activity was suppressed, and cancer cells were induced to increase apoptosis and promoted chemosensitivity to 5-FU and cisplatin (Hartojo et al., 2010). Consistent with this result, another study demonstrated that a reduction in cell viability in esophageal Eca109 and EC9706 cell lines treated with curcumin was associated with suppression of NF-κB (Tian et al., 2012a). Rawat et al. (2012) also showed that curcumin could inhibit bile acid-induced NF-κB activity and DNA damage in esophageal OE33 cell lines by inducing apoptosis. In addition, with the inhibition of the associated Rac1-PI3K-Akt signaling complex by curcumin treatment, the migration and invasion potential of SDF-1α-induced EC cells was attenuated (Lin et al., 2014). The PI3K/Akt pathway was demonstrated to induce oncogenic effects by activating Rac1 (Qiu et al., 1995). Curcumin-induced apoptosis in breast cancer cell lines was also promoted upon the suppression of the PI3K/Akt survival pathway (Kizhakkayil et al., 2010). Moreover, Liu et al. (2018b) reported that curcumin could suppress the JAK/STAT pathway to inhibit cell growth and induce apoptosis in ESCC. The authors further revealed the effect of curcumin on ESCC xenografts and showed that there was less tumor growth in these models. The JAK/STAT3 pathway was also related to cell cancer invasion and metastasis. Curcumin could enhance cell-cell adhesion and cell-matrix adhesion and reduce oxidative stress in esophageal Eca-109 cell lines by suppressing the JAK/STAT3 pathway, which helped reduce cell migration (Zheng et al., 2018). Furthermore, Notch signaling was suggested as a novel mechanism underlying the apoptosis-inducing effect of curcumin on EC cell lines (TE-7, TE-10, and ESO-1) (Subramaniam et al., 2012). The downregulation of Notch signaling reduced cell proliferation and colony formation and decreased the number of esophagospheres. The expression of Notch-1-specific microRNAs (miR-21 and miR-34a) was downregulated and that of tumor suppressor microRNA was upregulated in response to curcumin treatment. In conclusion, curcumin is an effective anticancer agent that induces the *in vitro* inhibition of EC cell lines by targeting different signaling pathways.

**TABLE 1:** *In vitro* studies for curcumin effects on EC.

Cell lines	Agents	Concentration	Effects	Mechanisms	Reference
Ec109	Curcumin	5, 10, 20, 40, and 80 μM	Suppressed cell growth and cell cycle arrest in the G2/M phase	Downregulation of glycolytic enzymes by activating AMPK	Zhang et al. (2015)
OE33	Curcumin	50 µM	Abrogated bile-induced DNA damage	Downregulation of NF-κB	Rawat et al. (2012)
CE 48T/VGH, CE 81T/VGH, and CE 146T/VGH	Curcumin	4 and 8 µM	Attenuated SDF-1α-induced cell migration and invasion	Inhibition of the associated Rac1-PI3K-Akt, the localization of CXCR4, and the expression of MMP-2	Lin et al. (2014)
EC1, EC9706, KYSE450 and TE13	Curcumin	4, 8, 12, and 16 µM	Inhibited cell growth and increased apoptosis	Downregulation JAK/STAT	Liu et al. (2018b)
Eca-109	Curcumin	5, 20 and 50 μM	Enhanced cell-cell adhesion and cell-matrix adhesion	Reduction of estrogen receptors-induced oxidative stress through downregulation of JAK/STAT3	Zheng et al. (2018)
TE-7, TE-10, and ESO-1	Curcumin	10, 20, 30, 40, and 50 μM	Reduced cell proliferation, colony formation, and increased apoptosis	Suppression of Notch by downregulating the secretase complex	Subramaniam et al. (2012)
KYSE30	2,6-bis benzylidine cyclohexanone analogues	0.015, 0.061, 0.244, 0.976, 3.906, 15.625, 62.5, 250, and 1000 μg/ml	Lower IC50 values, increased apoptosis and cell cycle arrest at G1 phase	N/A	Alibeiki et al. (2017)
SLMT-1, HKESC-2, and KYSE-270	Curcumin, and its analogues	Curcumin (5, 10, 15, and 20 µM) analogues (1, 2, 3, 4, and 5 µM)	Increased apoptosis and cell cycle arrest	N/A	Tung et al. (2018)
Eca109 and EC9706	2-pyridyl cyclohexanone analogues	0.8, 1.6, and 3.2 µM	Enhanced growth inhibition and apoptosis	Inhibition of the JAK2-STAT3	Wang et al. (2018)
TE-5, TE-8 and TE-11R	Theracurmin	25 and 50 µM	Decreased cell viability and inhibited spheroid formation and colony formation (TE-8 and TE-11R cells)	Increased intracellular ROS levels through activation of NRF2-NMRAL2P-NQO1	Mizumoto et al. (2019)
OE33, and OE19	Theracurmin	50 µM	Decreased cell proliferation and enhanced T cell induced cytotoxicity	Promotion of the immune system function against tumor cells	Milano et al. (2013)
TE-8 and SKGT-4	Curcumin, EGCG, and lovastatin	Curcumin (40 μmol/L), lovastatin (4 μmol/L), and EGCG (40 μmol/L)	Reduced cell viability and invasion capacity	Suppression of Erk1/2, c-Jun and COX-2 expression	Ye et al. (2012)
Eca109 and EC9706	Curcumin and 5-FU	50 µM	Decreased cell viability and enhanced chemosensitivity to 5-FU	Downregulation of NF-κB	Tian et al. (2012a)
Flo-1 and OE33	Curcumin, 5-FU, and cisplatin	6.25, 12.5, 25, and 50 μM	Increased apoptosis and promoted chemosensitivity to 5-FU and cisplatin	Downregulation of NF-κB	Hartojo et al. (2010)
Eca109 and EC9706	Curcumin, p65 siRNA, and 5-FU	50 µM	Reduced cell viability, higher apoptosis, and enhanced sensitivity to 5-FU	Suppression of NF-κB	Tian et al. (2012b)
TE-1	Nanoparticles carrier of recombinant HGFI and curcumin	1, 2, 4, 8, 16, and 32 μg/ml	Reduced cell viability, enhanced solubility and release of curcumin	N/A	Niu et al. (2020)
OE-19 and BAR-T (Barrett’s epithelium cell line)	Biopolymeric carrier of PLGA-b-PEG copolymer delivered with gold nanorods and curcumin	40 μM in gold and 166 μM in curcumin	Reduced cell viability	N/A	Martin et al. (2015)
KYSE150, and KYSE510	PH-responsive nanoparticle carrier loaded docetaxel and curcumin	N/A	Reduced cell viability, increased cellular uptake	N/A	Deng et al. (2020)

EGCG: (-)-epigallocatechin-3-gallate; COX-2: cyclooxygenase-2; MMP-2: matrix metalloproteinases-2; IC50: half maximal inhibitory concentration; ROS: reactive oxygen species; 5-FU: 5-fluorouracil; N/A: not applicable.

### 3.2 *In vivo* studies

In addition to evidence from *in vitro* studies, evidence from *in vivo* studies has also demonstrated the anticancer potential of curcumin (Table 2). Ushida et al. (2000) established a rat model of EC treated with N-nitrosomethylbenzylamine (NMBA) and evaluated the incidence of EC and moderate or severe epithelial dysplasia in the different groups. The group with the dietary feeding of curcumin showed a lower rate of EC and epithelial dysplasia, suggesting the inhibitory effect of curcumin on NMBA-induced esophageal tumorigenesis and cell proliferation. In an *in vivo* study conducted by Tian et al. (2012b), mice were treated with curcumin, p65 siRNA, and 5-FU after being implanted with esophageal EC9706 cells. The authors found that the size and weight of the tumors were significantly reduced in the curcumin+5-FU group and p65 siRNA+5-FU group compared to that in the group treated with 5-FU alone. Curcumin and p65 siRNA can potentiate the apoptotic effect of 5-FU in tumor tissues. In addition, the findings of this *in vivo* study revealed that the anti-cancer effect of curcumin was also achieved through the blockade of the NF-κB pathway. Furthermore, in a small pilot trial involving 33 patients with Barrett’s esophagus, 16 patients received oral curcumin tablets, and biopsy samples were collected from all patients (Rawat et al., 2012). An increased apoptotic frequency was observed in the epithelial cells of Barrett’s tissue from the group treated with curcumin, indicating that curcumin may confer beneficial effects on patients with Barrett’s esophagus.

**TABLE 2:** *In vivo* studies for curcumin effects on EC.

Animal models	Agents	Concentration	Effects	Mechanisms	Reference
Male F344 rats	Curcumin	Diet: 500 ppm	A lower rate of NMBA-induced EC and epithelial dysplasia	N/A	Ushida et al. (2000)
C57BL/6 male mice	Theracurmin	Diet: 2 g/kg containing 0.6 g/kg curcumin	Reduced tumor volume	Increased intracellular ROS levels through activation of NRF2-NMRAL2P-NQO1 pathway	Mizumoto et al. (2019)
Male athymic BALB/c nude mice	Curcumin and 5-FU	50 µM	Decreased tumor volume	Downregulation of NF-κB	Tian et al. (2012a)
Male athymic BALB/c nude mice	Curcumin, p65 siRNA, and 5-FU	50 µM	Reduced size and weight of tumor	Suppression of NF-κB	Tian et al. (2012b)
Female CB17/SCID mice	Curcumin, and 5-FU	Injection: 100 mg/kg	Reduced weight of tumor	Downregulation of JAK/STAT	Liu et al. (2018b)
Male nude mice	Curcumin, and analogue SSC-5	Both 50 mg/kg for curcumin, and analogue SSC-5	Reduced tumor size and tumor invasion	N/A	Tung et al. (2018)
Nu nude mice	Curcumin, EGCG, and lovastatin	All 50 μg/kg for curcumin, EGCG, and lovastatin	Suppressed tumor growth	Suppression of Erk1/2, c-Jun and COX-2 expression	Ye et al. (2012)
Rat model of Barrett’s esophagus	Biopolymeric carrier of PLGA-b-PEG copolymer delivered with gold nanorods and curcumin	0.3 ml by endoscopy injection	Elimination of the mucosa destruction of Barrett’s esophagus	N/A	Martin et al. (2015)
Male Balb/c mice	PH-responsive nanoparticle carrier loaded docetaxel and curcumin	Injection	Reduced tumor volume	N/A	Deng et al. (2020)

EGCG: (-)-epigallocatechin-3-gallate; COX-2: cyclooxygenase-2; ROS: reactive oxygen species; 5-FU: 5-fluorouracil; N/A: not applicable.

### 3.3 Clinical trials

Clinical trials have been conducted on the therapeutic effects of curcumin in different cancers. Unfortunately, no clinical trial has investigated the effectiveness of curcumin alone in EC.

A clinical randomized study assessed the anticancer effect of curcumin on CRC cells in 126 patients (He et al., 2011). Half of the patients received oral curcumin capsules at a dose of 360 mg/day before surgery, and these patients showed an increased body weight and decreased serum levels of tumor necrosis factor-α (TNF-α) in response to curcumin treatment. TNF-α is an inflammatory cytokine released by cancer cells, which contributes to malignant progression (Balkwill, 2006). Besides, a greater number of apoptotic cells, DNA fragmentation, and p53 expression were observed in CRC tissues in the curcumin-treated group compared to that in the control group. P53 acts as an apoptosis inducer and tumor suppressor by modulating apoptotic pathways (Teodoro et al., 2006). Curcumin induced cancer cell apoptosis by enhancing p53 expression. Therefore, curcumin supplementation may be safe and effective for patients with CRC. A phase II clinical trial enrolling 25 patients with advanced PC demonstrated that curcumin was well tolerated and exhibited no toxic effect when administered at 8 g/day until disease progression (Dhillon et al., 2008). The expression of some markers associated with tumor development (NF-κB, cyclooxygenase-2, and pSTAT3) was reduced in peripheral blood samples from patients compared to that in samples collected from healthy participants. In addition, limited biological activity of curcumin and its potential anticancer effect were observed in two patients. This evidence showed the potential therapeutic role of curcumin in the treatment of PC. Data from a recent prospective phase II trial suggested that patients receiving gemcitabine supplemented with the phytosome complex of curcumin for advanced PC had an overall survival of 10.2 months, which was greater than that reported in previous studies on patients treated with gemcitabine alone (Pastorelli et al., 2018). The curcumin complex can enhance the chemotherapeutic efficacy of gemcitabine in advanced PC and is safe and beneficial.

## 4 Improvement of the anticancer efficacy of curcumin

### 4.1 Analogs of curcumin

Despite the promising outcomes of curcumin against cancer, the clinical utility of curcumin is limited owing to its low solubility in water and poor absorption. New analogs are being developed by structural modification for better efficacy, which can help preserve the primary activity of curcumin and promote its stability and bioavailability. The conjugates of curcumin with some amino acids show greater solubility, promote cellular uptake, and lower metabolic degradation, which leads to stronger anticancer activity (Dubey et al., 2008). The carrier proteins of amino acids can carry additional curcumin molecules into the cell, thus enhancing the accumulation of curcumin. Alibeiki et al. (2017) synthesized 2,6-bis benzylidine cyclohexanone analogs of curcumin and investigated the anticancer mechanism of action of these analogs in GC and EC cell lines. These analogs caused the upregulation of apoptosis-related factors (Bax and caspase-3) and downregulation of anti-apoptotic factors, indicating that the analogs triggered the apoptotic pathway. Compared to curcumin, the novel synthesized analogs showed greater potency in the inhibition of cancer cell proliferation, apoptosis induction, and cell cycle arrest. In an *in vitro* study by Tung et al. (2018), eight newly synthesized curcumin analogs exhibited better anti-tumor effect in EC cells than curcumin. The authors further developed tumor xenograft models in mice, and analog SSC-5 was selected for the subsequent *in vivo* study because it had the strongest ability to induce apoptosis and cause cell cycle arrest. The tumor size and risk of tumor invasion were reduced in xenograft mice treated with SSC-5. Moreover, the anticancer effect on and molecular mechanisms of action of 2-pyridyl cyclohexanone analogs of curcumin in esophageal Eca109 and EC9706 cells were also explored (Wang et al., 2018). These analogs could inhibit cell growth and induce cell apoptosis by downregulating STAT3 phosphorylation and Bcl-2 expression.

### 4.2 Combination of curcumin with other drugs or therapeutic agents

A new formulation of curcumin, named Theracurmin, was demonstrated to exhibit stronger inhibitory effect on the proliferation of EC cells and the growth of xenografted tumors than curcumin, owing to its high bioavailability (Mizumoto et al., 2019). Curcumin is present in the form of colloidal submicron particles in Theracurmin. Besides, combined treatment with Theracurmin and an NAD(P)H quinone dehydrogenase (NQO1) inhibitor showed better anticancer effectiveness by regulating the NRF2-NMRAL2P-NQO1 pathway. NQO1 protected cells from oxidative stress and functioned as an antioxidant in Theracurmin -mediated cytotoxicity. NQO1 inhibitor can increase oxidative stress and enhance the sensitivity of cells to Theracurmin, thus promoting the anticancer effect. Moreover, Theracurmin was shown to inhibit the proliferation of EC cells but exerted no effect on normal esophageal cells, which indicates the safety of Theracurmin (Milano et al., 2013). Dendritic cell (DC)-based immunotherapy seems to be a promising treatment for cancer. DC loaded with tumor antigens was prepared *ex vivo* and delivered to patients to activate their immune system (Milano et al., 2007). The authors suggested that the combination of DC immunotherapy with Theracurmin is effective for the treatment of EC (Milano et al., 2013). Theracurmin can enhance the anticancer response of the immune system by promoting the function of DCs and T cells and sensitizing cancer cells to cytotoxicity. Statins and (-)-epigallocatechin-3-gallate (EGCG) were shown to suppress tumorigenesis in esophageal cancer (Li et al., 2002; Ogunwobi and Beales, 2008). Ye et al. (2012) applied curcumin, lovastatin, and EGCG and investigated their combined effects in EC cell lines and mouse xenografts. The authors found that cell viability and tumor growth were inhibited upon the combined treatment *via* the downregulation of Erk1/2, c-Jun, and cyclooxygenase-2 expression. However, possibly owing to the low water solubility of curcumin, it exerted no effect on tumor formation and growth in mice treated with the highest dose of curcumin alone. Therefore, the application of combination therapy is useful for improving the synergistic effects of drugs for better therapeutic efficacy. The interaction between curcumin and other drugs warrants additional investigation.

### 4.3 Application of novel nanocarriers

The use of novel delivery systems formulated using different types of biopolymers is of great interest for the application of curcumin *via* nanocarrier particles. Biopolymeric carriers can enhance the stability and bioavailability of biopolymer molecules through the targeted and sustained release of their components. A new nanoparticle carrier of recombinant HGFI and curcumin was designed and exhibited higher solubility and stability in aqueous solution for up to 12 h (Niu et al., 2020). Besides, the nanoparticles promoted the release of curcumin and enhanced cytotoxicity against EC cells compared to free curcumin; this effect was attributed to the amphiphilic property and self-assembly characteristics of HGFI. Hydrophobin HGFI is an amphiphilic protein, capable of forming amphipathic membranes through self-assembly at hydrophilic-hydrophobic interfaces (Szilvay et al., 2007). The authors also suggested that HGFI coating was an effective method for preparing nanoparticles. Martin et al. (2015) developed a biopolymeric carrier of a PLGA-b-PEG copolymer delivered with gold nanorods and curcumin and investigated its effect on Barrett’s esophagus and EC. The results showed a significant decrease in cell viability in Barrett’s esophagus and cancer cell lines but no significant changes in benign esophageal cell lines. *In vivo* investigation revealed that the destruction of the mucosa in Barrett’s esophagus was eliminated in response to the synergistic toxic effect of gold nanorods and curcumin. Gold nanorods can cause damage to cancer cells by inducing hyperthermia through the photothermal effect after laser light exposure (Chen et al., 2007). This novel biopolymeric carrier could deliver gold nanorods and curcumin to the target tumor site to exert a synergistic anticancer effect without influencing the adjacent normal tissue. Stimuli-responsive nanoparticles can control drug release by responding to the differences between normal and tumor tissues. The pH-responsive nanoparticle polymers can sense the change in the acidity of the tumor microenvironment and selectively release the contained drug at the tumor site, thus eliciting effective anticancer activity (Xie et al., 2018). Similarly, Deng et al. (2020) designed a pH-responsive nanoparticle carrier that improved the anticancer effects of docetaxel and curcumin in EC. In addition, this nanoparticle carrier was modified with a T7 peptide and promoted the permeability and retention of curcumin and increased the cellular uptake efficiency.

Nanoparticle structure modification constitutes a new direction in this field. For instance, cell membrane wrapping is a new strategy for the modification of nanoparticles that exhibit different properties owing to the presence of different types of cell membranes. Nanoparticles wrapped with the tumor cell membrane can achieve the homologous recognition of tumor cells and are more precisely targeted to tumors (Zhen et al., 2019). MDR in tumors is a major obstacle to clinical chemotherapy and severely affects the effectiveness of chemotherapy. MDR is characterized by the resistance of tumor cells to multiple drugs with different structures and mechanisms of action, which makes it difficult for anticancer drugs to reach effective intracellular concentrations. Gao et al. (2021) used esophageal TE10 cell membrane to encapsulate PLGA-nanoparticles loaded with curcumin and doxorubicin and investigated the therapeutic efficacy of this combination in the treatment of drug-resistant EC. TE10 cell lines were established to elicit resistance to doxorubicin and were then used in a mouse model. The results confirmed that this new nanoparticle carrier showed controlled drug release, targeted intracellular uptake, and high biological safety, thus achieving efficient drug delivery and enhancing the anticancer efficacy in MDR cancer cells.

Different types and structures of biopolymers can affect the stability and bioavailability of curcumin, leading to varied drug efficacy. The designing of new nanoparticle carriers of curcumin combined with multiple drugs integrating the functions of controlled drug release and targeted action on tumor location can broaden the scope of curcumin application in tumor treatment. Furthermore, the modification of the nanoparticle structure and encapsulation of curcumin also show promise in clinical application.

## 5 Discussion

The anticancer effect of phytochemicals such as curcumin has been demonstrated in the treatment of various cancers, especially in cancers of the gastrointestinal system. The esophagus, which is connected to the pharynx and the cardia of the stomach, is the necessary passage for food digestion and absorption. When various conditions and diseases develop in the esophagus, the patient experiences severe pain, and treatment is complicated because of its physiological location, structure, and functions. Additionally, the incidence of EC and precancerous conditions such as Barrett’s esophagus is increasing, which poses a major threat to human health. Owing to the low bioavailability of orally administered curcumin, EC seems to be more suitable for curcumin treatment than other non-gastrointestinal cancers. In fact, the application of curcumin has promising outcomes in the treatment of EC.

The specific anticancer mechanism of action of curcumin is not fully understood. Curcumin inhibits tumor cell growth, migration, invasion, and angiogenesis and promotes apoptosis through the regulation of various cellular molecules, growth factors, miRNAs, and genes involved in different signaling pathways. Besides, curcumin can sensitize chemoresistant and radioresistant cancer cells, reversing their resistance and making them more susceptible to inhibition or death. Curcumin exhibits both direct and indirect anti-EC activity in different cell lines and animal models, which indicates its potential as a phytochemical agent in the prevention and treatment of EC. To better increase the bioavailability and efficacy of curcumin, strategies such as the modification of curcumin analogs, combination with other drugs or therapies, and use of novel nanocarriers have been explored extensively.

The effect and bioavailability of curcumin is determined by its treatment period, concentration, and the type of cells. A limitation in the application of curcumin is its low bioavailability. The synthesis of new curcumin analogs has recently garnered significant interest in research. The new analogs not only retain the original properties of curcumin but also enhance its potency and stability owing to the modification of different side chain groups.

Radiotherapy and chemotherapy are important modalities in the treatment of EC, whereas resistant cancer cells may contribute to the failure of treatment. The combination of traditional chemical agents or radiation with natural phytochemicals is an important strategy in this field, where curcumin exhibits superior sensitizing effects. The sensitizing potential of curcumin can be used to achieve superior therapeutic effects and reduce the adverse effects of treatment by aiding the minimization of the dose of chemotherapeutic drugs or radiation. Besides, curcumin in combination with other anticancer agents may improve the synergistic effect of drugs and enhance their efficacy.

Nanocarriers can be efficiently loaded with multiple drugs simultaneously, and their surfaces can be functionally modified. Different modifications can lead to a longer *in vivo* circulation time of the carrier, lower drug toxicity, and greater bioavailability of the drug. The nanocarrier surface can also be linked to tumor-specific ligands to enhance the targeting of drugs and reduce the damage to normal tissues.

The above findings give us a glimpse of the benefits of curcumin in the treatment of cancer, or EC in particular. Although curcumin is safe, it is unknown whether its long-term use can cause any side effects. However, the anticancer effects of curcumin have been observed more frequently in cellular and animal models, and more convincing clinical trials need to be conducted to confirm its effectiveness. For these results to be used in clinical antitumor therapy, further studies on the effects, mechanisms, absorption, and metabolic characteristics of curcumin and its analogs, combined therapies, and new nanocarrier forms in humans are required. Therefore, we have reason to believe that curcumin, an ancient phytochemical, will play a prominent role in the prevention and treatment of EC in the future.
